# A novel approach to pulsed laser deposition of platinum catalyst on carbon particles for use in polymer electrolyte membrane fuel cells

**DOI:** 10.3762/bjnano.14.19

**Published:** 2023-02-02

**Authors:** Bogusław Budner, Wojciech Tokarz, Sławomir Dyjak, Andrzej Czerwiński, Bartosz Bartosewicz, Bartłomiej Jankiewicz

**Affiliations:** 1 Institute of Optoelectronics, Military University of Technology, 2 Kaliskiego Str., 00-908 Warsaw, Poland; 2 Łukasiewicz Research Network - Mościcki Industrial Chemistry Research Institute (ICRI), 8 Rydygiera Str., 01-793 Warsaw, Poland; 3 Institute of Chemistry, Military University of Technology, 2 Kaliskiego Str., 00-908 Warsaw, Poland; 4 Faculty of Chemistry, University of Warsaw, 1 Pasteura Str., 02-093 Warsaw, Poland

**Keywords:** carbon particles, cyclic voltammetry, fuel cells, ORR, PEMFCs, PLD deposition, Pt catalyst, rotating ring-disk electrode (RRDE), SEM, TEM, XPS

## Abstract

The research undertaken aimed to develop an efficient Pt-based catalyst for polymer electrolyte membrane fuel cells (PEMFCs) by using a cost-effective and efficient physical method to deposit platinum nanoparticles (PtNPs) on carbon supports directly from the platinum target. The method developed avoids the chemical functionalization of the carbon substrate and the chemical synthesis of PtNPs during catalyst fabrication. Platinum was deposited on carbon particles at room temperature using a pulsed laser deposition (PLD) system equipped with an ArF excimer laser (λ = 193 nm). The uniform deposition of PtNPs on carbon supports was achieved thanks to a specially designed electromechanical system that mixed the carbon support particles during platinum deposition. In the studies, Vulcan XC-72R carbon black powder, a popular material used as support in the anodes and cathodes of PEMFCs, and a porous carbon material with a high degree of graphitization were used as carbon supports. The best electrochemical measurement results were obtained for Pt deposited on Vulcan XC-72R. The peak power density measured for this material in a membrane electrode assembly (MEA) of a PEMFC (fed with H_2_/Air) was 0.41 W/cm^2^, which is a good result compared to 0.57 W/cm^2^ obtained for commercial 20% Pt Vulcan XC-72R. This result was achieved with three times less Pt catalyst on the carbon support compared to the commercial catalyst, which means that a higher catalyst utilization factor was achieved.

## Introduction

Fuel cells, which cleanly and efficiently convert the chemical energy of hydrogen or other fuels to electrical energy, are a good alternative to dirty and wasteful combustion engines for electric power generation. Among various fuel cells, polymer electrolyte membrane fuel cells (PEMFCs) have received considerable attention because of several physicochemical advantages over other fuel cell types [1–5]. PEMFCs, constructed of polymer electrolyte membranes as the proton conductor and an electrochemical catalyst for electrochemical reactions under low temperatures, are typically used in automobiles and portable electronics [4–5]. The primary application potential is related to their compact size, lightweight, high power density, and low operating temperature. However, they also have some limitations that prevent their wider use [3–4]. Two of the significant limitations of PEMFCs are durability and cost, which are related to each other in an unfortunate way because durability decreases as the loading of the costly electrocatalyst is reduced [1–3].

Both durability and cost of PEMFCs depend on the materials used to construct their major components, which are anode, cathode, and polymer electrolyte membranes [3,6]. Therefore, supplying good-performance materials with controlled nanostructures to fuel cell technology is a crucial issue [7]. One solution to this problem is to use material nanoarchitectonics, a new concept that emerged several years ago and has become widely applicable to material science [8–10]. Fabricating functional materials by combining two or more existing materials into one exhibiting unusual and often unpredicted properties is a powerful strategy for materials creation by nanoarchitectonics [10]. Nanoarchitectonics can be used to design and fabricate innovative catalysts by tailoring their molecular composition, surface atomic arrangement, and microstructures [11]. However, it requires harmonizing various techniques and phenomena [12–14]. Recently, nanoarchitectonics approaches have been used to fabricate various materials for energy-related applications, including carbon-based composites [15–16], Pt-based nanostructures and composites with carbon materials [17–19], and metal alloys deposited on TiO_2_ [20].

The most commonly used catalyst in PEMFCs is platinum on various carbon support materials, which is used in both the anode and cathode because of its high catalytic activity toward the hydrogen oxidation reaction (HOR) and oxygen reduction reaction (ORR) [6,17–19,21–25]. Pt is also characterized by its durability in acidic environments under high potential [23]. However, platinum, which has to be used in PEMFCs in relatively significant amounts, is also scarce, expensive, and sensitive to CO poisoning at standard operating temperatures [4]. Therefore, many research studies have been dedicated to reducing the Pt loading at the cathode without performance loss of PEMFCs or to finding an alternative catalyst material [4,6,21]. The reduction of Pt loading on various carbon supports can be achieved by combining Pt with other metals [21] as well as by modifications of the various chemical and physical methods of Pt deposition [22–26]. Direct deposition of Pt onto carbon supports resulting in a thin catalyst layer and good dispersion of formed Pt nanoparticles (PtNPs) is of particular interest because it should allow for a high Pt mass-specific power density to be achieved [27]. Direct deposition of PtNPs can be attained by the use of various physical vapor deposition techniques such as magnetron sputtering [28], sputtering [29], e-beam evaporation [30], dual ion-beam assisted deposition [31], and pulsed laser deposition (PLD) [27,32–33]. Previously, PLD has been used to deposit Pt for the cathode of PEM fuel cells only in a few studies, and in all of them only on gas diffusion layers of carbon fabric or on proton conductive Nafion membrane [27,32–33]. According to our knowledge, no studies have been reported on the PLD deposition of Pt catalyst on carbon supports in the form of nano- and microparticles.

Herein, we report on the deposition of platinum on carbon particles using PLD to fabricate cost-efficient catalysts for PEMFCs with good performance. The research aimed to develop an effective physical method of PtNP deposition on carbon supports directly from the platinum target to eliminate the chemical functionalization of the carbon substrate and the chemical synthesis of PtNPs. The Pt catalyst was deposited on synthesized highly graphitized carbon particles and XC-72R commercial carbon support using PLD with a specially designed electromechanical system for carbon support mixing during PLD deposition. The highly graphitized carbon particles used as support were synthesized using metallothermic reduction. We additionally investigated the influence of deposition process parameters on the morphology of the fabricated Pt/C catalysts. Structure, morphology, and chemical composition of the fabricated catalysts were investigated using TEM, SEM, EDX, XPS, and Raman spectroscopy. Electrochemical measurements determined the performance of the fabricated catalysts.

## Results and Discussion

### Synthesis of a highly graphitized carbon material

**Figure 1:**
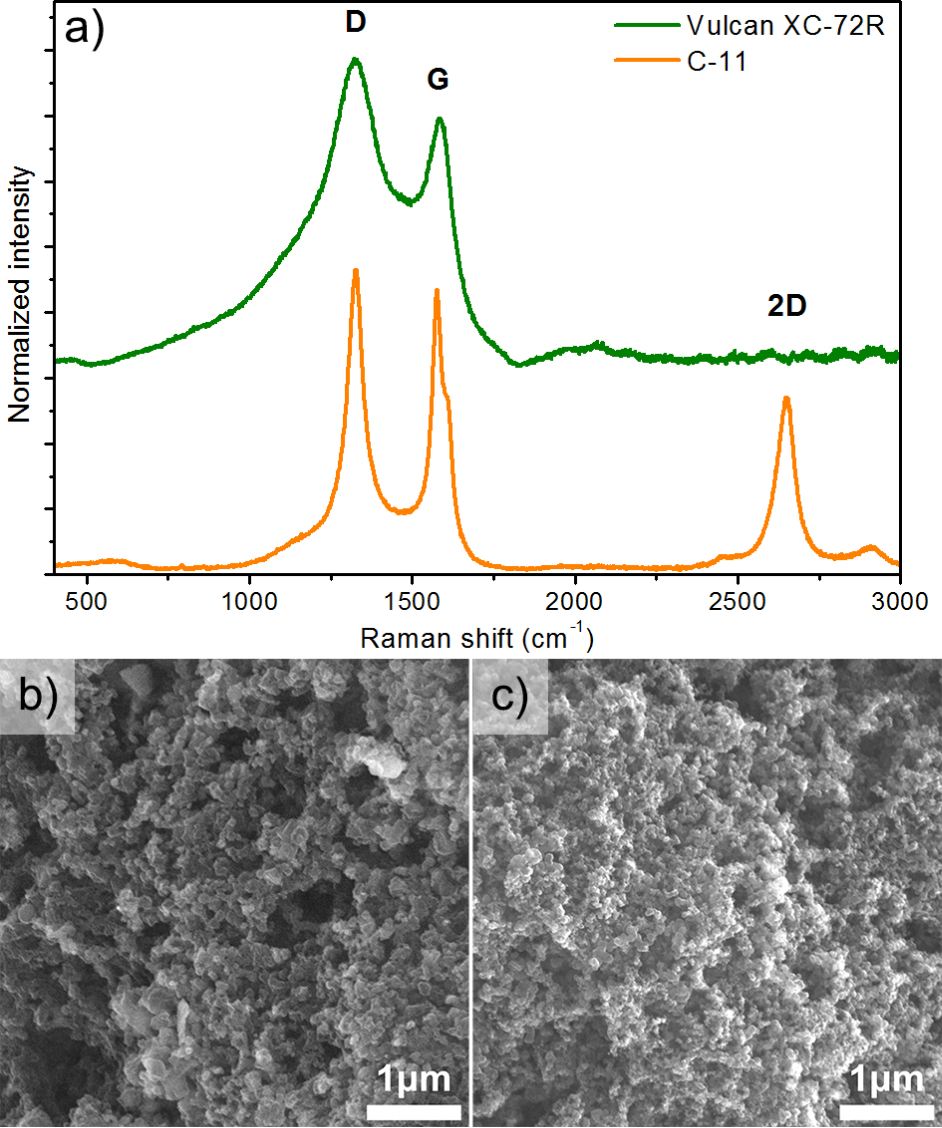
(a) Raman spectra of carbon supports, commercial Vulcan XC-72R, and synthesized C-11, (b) SEM images of the synthesized carbon material C-11, and (c) SEM image of the Vulcan XC-72R reference material.

The synthesis of the highly graphitized carbon material was carried out using a method similar to the methods described in detail in earlier works [34–35]. The synthesis of carbon material was carried out in a high-pressure stainless steel reactor with an internal volume of 1 dm^3^. It was initiated under 1 bar (absolute) pressure in an argon atmosphere. After thermal ignition, a mixture of magnesium and calcium formate powders (mixed with a 6:1 molar ratio) reacted vigorously in a self-propagating high-temperature regime, giving rise to MgO/CaO and carbon as the main solid-state products. After the reaction, a dark-grey powder was collected from the crucible and purified by stirring in an aqueous solution of hydrochloric acid (20 wt %) at boiling point (to remove MgO, CaO, and other byproducts of the combustion soluble in acids). After acid treatment, the black powder was filtered, washed with distilled water and acetone, and dried at 80 °C to constant weight. The black carbon product was referred to as a C-11. The research assumed the synthesis and use of a carbon material with a high degree of graphitization as carbon support, which is more resistant to the high-temperature oxidation process in a working fuel cell [36–38]. Using the combustion method under appropriate synthesis conditions allows for the fabrication of a very high content of highly graphitized carbon particles. However, the research showed that some materials form dense agglomerates with large dimensions and do not disperse sufficiently well, which is a problem for the surface deposition of catalysts and leads to ineffective operation in the fuel cell (poor O_2_ and H_2_ transport conditions). Considering the above factors, the material marked as C-11 was used as carbon support for the Pt catalyst due to its structure. This material consists of carbon particles with a mean diameter below 0.5 μm that form loosely arranged structures (Figure 1).

The comparison of Raman spectra of the synthesized carbon material C-11 and the commercial carbon support Vulcan XC-72R is shown in Figure 1. The differences in the crystalline structure of these materials are significant, as evidenced mainly by the differences in the width of the peaks and the ratio of D to G peak heights. The Vulcan material is not well-ordered (close to amorphous), as evidenced by the large width of the D and G peaks. In contrast, the spectrum of the C-11 material has narrow D and G peaks and an intense 2D band, which proves the highly graphitic structure. The D to G peak height ratio is 1.24 and 1.07, respectively, for the Vulcan XC-72R and C-11 material, which means that both materials have a high content of sp^3^-hybridized carbon.

**Figure 2:**
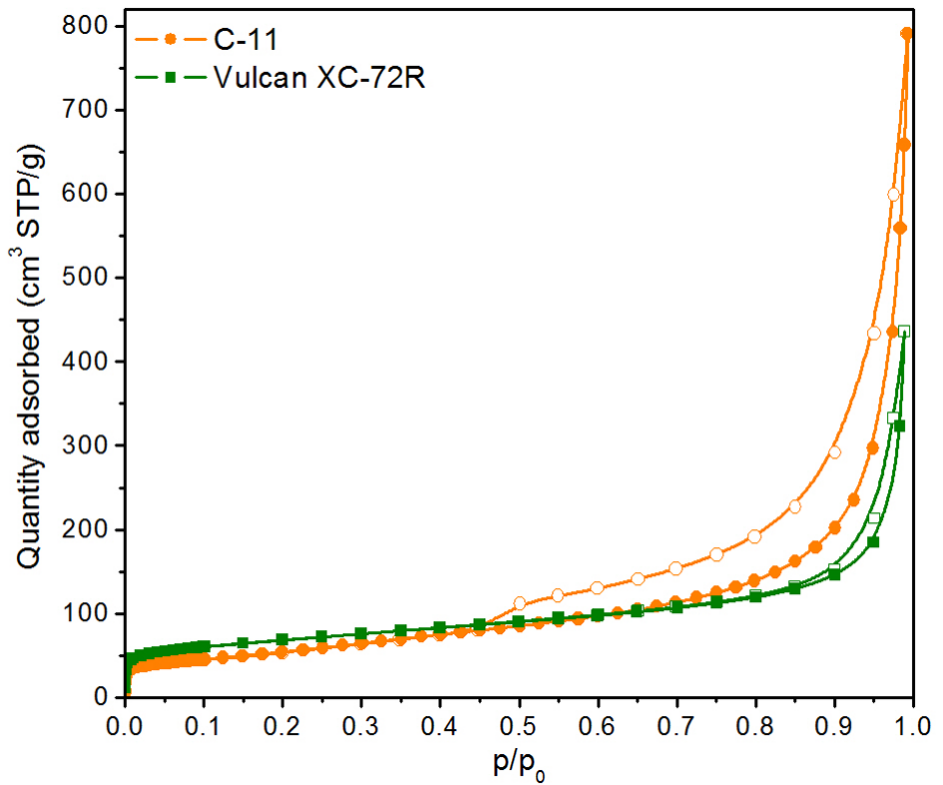
N_2_ adsorption–desorption curves for synthesized C-11 and commercial Vulcan XC-72R carbon materials.

Both carbon materials were also characterized in terms of their specific surface area (*S*_BET_) and pore volume. The N_2_ measured adsorption–desorption curves are shown in Figure 2. The *S*_BET_, calculated on their basis, is 204 m^2^/g for C-11 and 244 m^2^/g for Vulcan XC-72R. The calculated volume of micropores is 0.07 cm^3^/g for C-11 and 0.10 cm^3^/g for Vulcan XC-72R, while the total pore volume is 1.22 cm^3^/g for C-11 and 0.68 cm^3^/g for Vulcan XC-72R. Based on the above, Vulcan has a small fraction of micropores, although more significant than C-11, and a less developed mesoporosity than C-11. C-11 has depleted microporosity but more developed mesoporosity, i.e., a wider hysteresis loop and a higher isotherm with *p*/*p*_0_ values approaching 1. Both isotherms (i.e., of XC-72R and C-11) can be classified as type II (according to IUPAC classification [39]) but with H3-type hysteresis loops. A sharp increase of N_2_ adsorption capacity at *p*/*p*_0_ values approaching 1.0 is typical for particulate materials with extensive interparticle porosity and indicates the presence of large aggregates of particles. Such isotherms are typical for carbon black. As elucidated by Holdcroft et al. [40], the primary particles of carbon black materials coalesce into few-hundred-nanometer agglomerates. Within such agglomerates (up to 300 nm in size), mesopores of up to 20 nm diameter arise. Carbon black particle agglomerates merge into chainlike structures, forming a network of mesopores (>20 nm) in the interstices, which extend into interagglomerate macropores. In the case of carbon black-like materials, the primary carbon particles agglomerate into spherical agglomerates of ca. 200 nm and then merge into larger moieties of several micrometers, giving rise to an extensive meso–macroporous network. Such character of particle aggregation is observed for many types of carbon black materials [40]. It is important to note that the higher *S*_BET_ value for Vulcan XC-72R is solely due to its enhanced microporosity, yet its mesoporosity is significantly less developed compared to C-11. Therefore, the C-11 material is a better choice for deposition using the PLD method, because Pt will not enter the micropores but will be deposited in the meso- and macropores. Also, in a working fuel cell, extensive mesoporosity of a catalyst layer is more desired than microporosity since micropores remain mainly inaccessible to an ionomer and a catalyst.

### Deposition of platinum on a carbon support

Platinum was deposited on the synthesized carbon material C-11 and Vulcan XC-72R, one of the most popular materials used as catalyst support in the anode and cathode electrodes of PEMFCs. The platinum deposition was performed in vacuum at a pressure of around (4.6 ± 0.8) × 10^−5^ mbar. The PLD method allows one to control the amount of deposited platinum by changing the fluency of the laser radiation on the surface of the target or by changing the number of laser pulses. In these studies, the amount of deposited Pt was controlled only by changing the number of laser pulses. The Pt deposition was conducted with a number of laser pulses varying between 10000 and 60000 to investigate the influence of the amount of deposited platinum on the size of the formed PtNPs and their distribution on carbon supports (Table 1).

**Table 1:** Characteristics of catalysts obtained by the PLD deposition of platinum on carbon supports.

Sample designation	Carbon support	Number of laser pulses	Pt to C ratio determined from XPS	Pt to C ratio determined from EDS

A (XC/50k Pt)	XC-72R	50000	0.033	0.003
B (XC/60k Pt)	XC-72R	60000	0.056	0.005
C (CMg/10k Pt)	C-11	10000	0.087	0.002
D (CMg/40k Pt)	C-11	40000	0.210^a^	—
20% Pt XC-72R	—	—	0.035	0.016
HiSpec 3000	—	—	0.031	0.013

^a^Value estimated based on the XPS measurements for samples prepared with smaller and bigger numbers of laser pulses.

**Figure 3:**
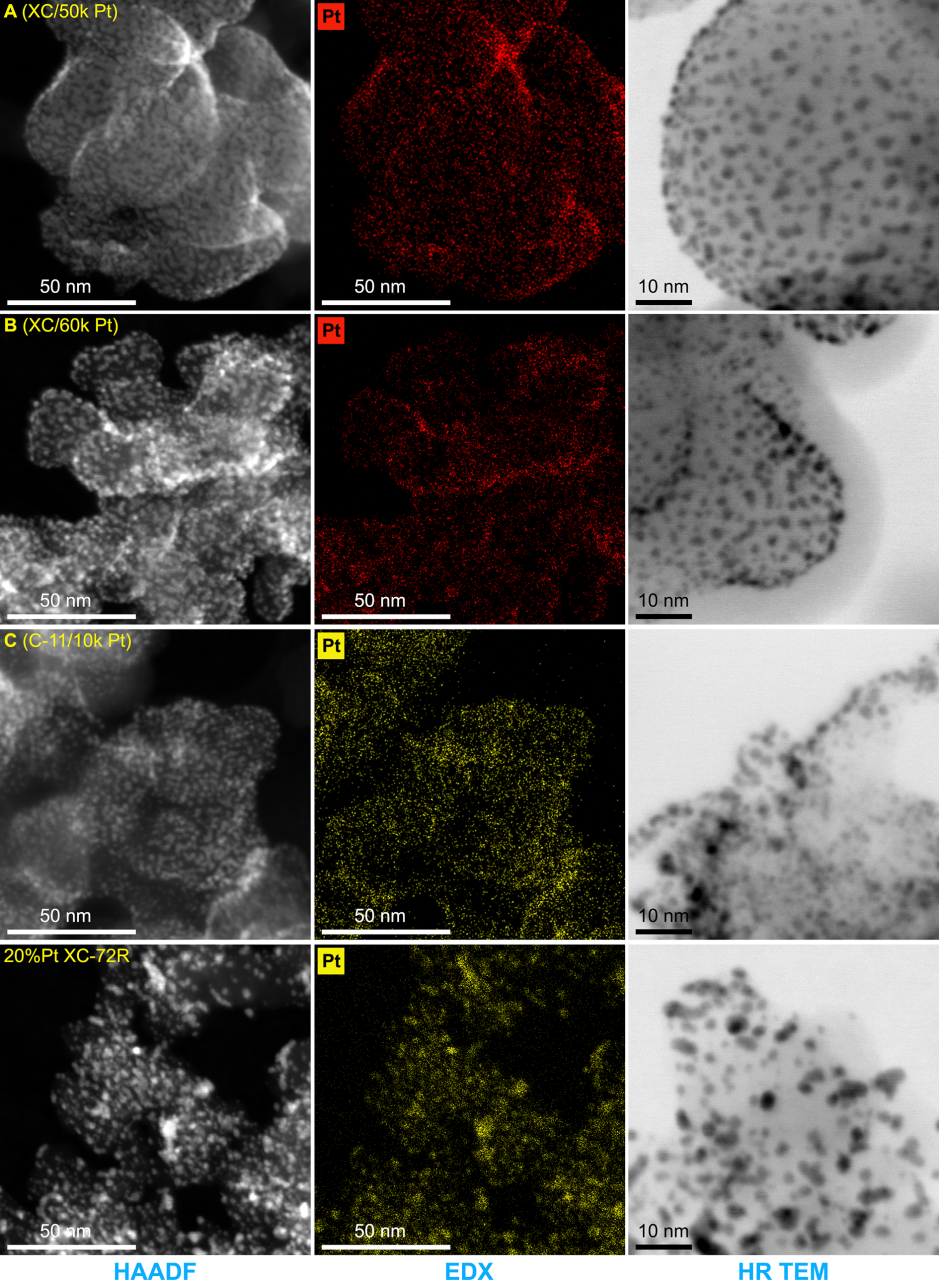
Results of TEM and EDX measurements of selected samples with Pt deposited using PLD and reference catalyst 20% Pt XC-72R. In the three columns, the results of HAADF, EDX, and HRTEM measurements are shown. Statistical analysis of Pt nanoparticle area distribution is shown in Supporting Information File 1, Figure S1.

The results of the TEM and EDX measurements are shown in Figure 3. Based on the HAADF-STEM images, it can be concluded that the distribution of PtNPs (white dots on images) is heterogeneous and that their sizes within one sample are very similar (samples A, B, and C). However, because the carbon particles are irregular 3D structures, the PtNPs appear larger and agglomerated on the edges and planes perpendicular to the imaging plane, although they can be above or below each other. Therefore, it can be concluded that the irregular PtNPs with dimensions of 1–4 nm are evenly deposited on the surface of carbon particles. Changing the number of laser pulses from 50000 to 60000 does not translate into noticeable differences in the PtNP dimensions. Based on the HAADF-STEM images, only a slight increase in the density distribution of the PtNPs is visible. The differences in the size of the PtNPs are better visible only between the samples prepared using 10000 and 60000 laser pulses. The differences in the dimensions of the PtNPs can be seen better in the images taken in the HRTEM mode.

Additional information is also provided by the statistical analysis of the PtNP area distribution based on the HRTEM images (Supporting Information File 1, Figure S1). This analysis was performed for the areas of the nanoparticles, because a number of the PtNPs have an irregular, elongated shape. In the case of sample A, PtNPs with an area of 2.5 to 4.5 nm^2^ dominate. In the case of sample B, PtNPs with an area of 3 to 5 nm^2^ predominate, and the fraction of particles with an area of more than 5 to 7 nm^2^ is increasing simultaneously. PtNPs with the smallest areas from 2.5 to 4.0 nm^2^ dominate in sample C. However, the mean area of PtNPs deposited by the PLD method for each sample is twice smaller than that of reference catalysts, where particles with an area of 5 to 11 nm^2^ are dominant. The most important observation from the TEM analysis is that the PLD method allows for the deposition of PtNPs with even smaller dimensions than the size of PtNPs in the reference catalyst and with a denser distribution. EDX images taken with a spatial resolution of 160 pm confirm the presence of PtNPs on the carbon support surface. Additionally, the EDX studies show that in the area of the edge of the carbon particles, the Pt signal is more intense, likely due to PtNPs overlapping, which looks like an agglomeration.

**Figure 4:**
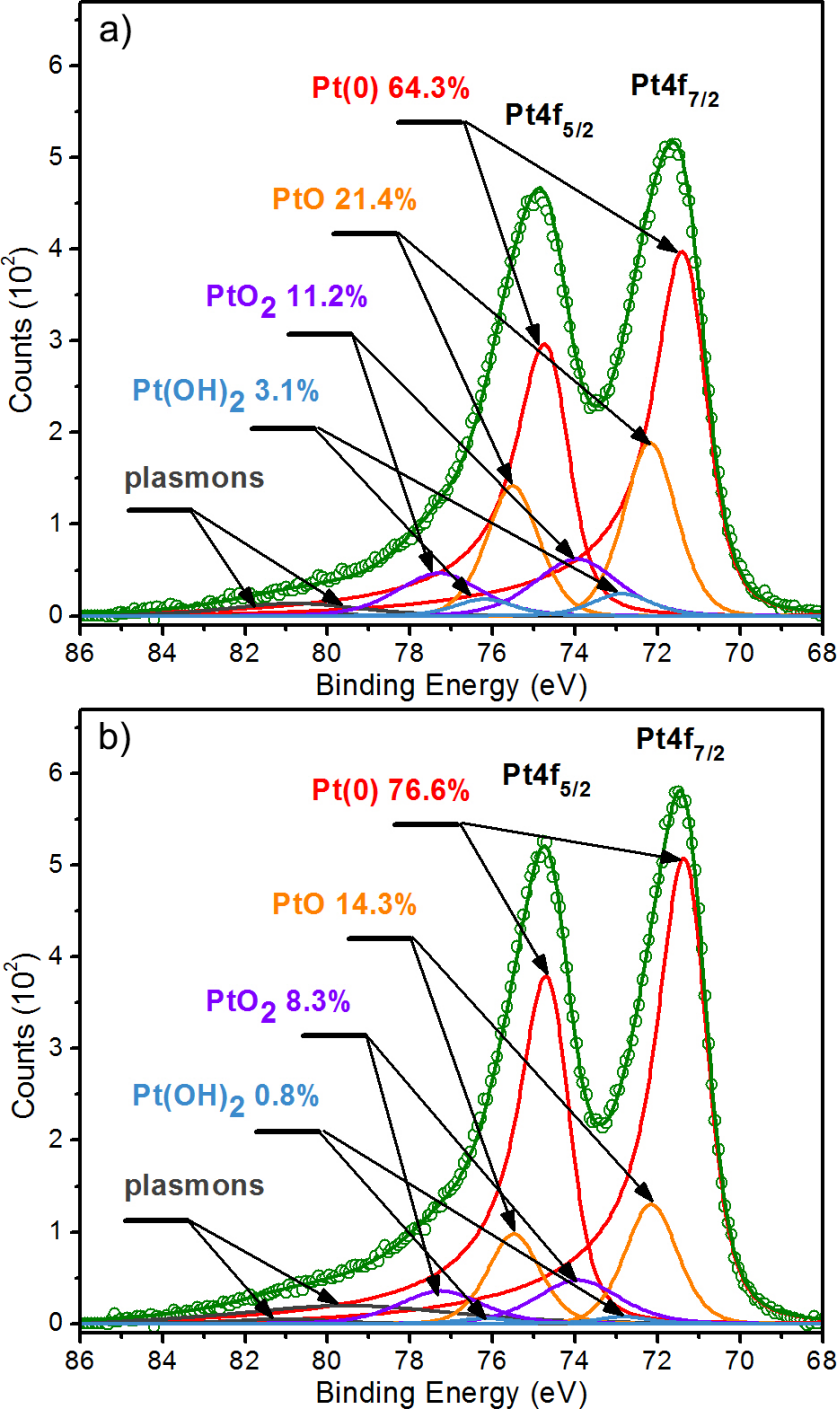
High-resolution XPS spectra of the Pt 4f band for (a) reference catalyst 20% Pt XC-72R and (b) sample A prepared using PLD characterized by a similar Pt-to-C ratio determined from XPS measurements.

XPS spectra in a wide binding energy range (Supporting Information File 1, Figure S2) were recorded from an area of about 1.5 × 3.5 mm for materials prepared in the form of layers with a thickness of about 0.4 mm. However, due to the small depth of collecting the photoelectrons in XPS (about 3 to 10 nm), the recorded signal comes only from the material’s surface. Consequently, the Pt content determined may be significantly influenced by the size of the PtNPs and their distribution on the sample surface. Namely, for the same weight fraction of platinum, the ratio of Pt-to-C in a sample with larger PtNPs (from 4 to 6 nm) located at large distances from each other should be noticeably lower than for a sample with very small PtNPs (from 1 to 3 nm) densely distributed on the surface of the carbon support. Nevertheless, this XPS technique feature can be used to infer whether the PtNPs cover the carbon particles uniformly. Therefore, XPS spectroscopy was used in our studies to determine the ratio of Pt-to-C (based on the atomic concentration of the elements) for comparing Pt coatings with reference materials (Table 1) and evaluating the quality of the deposited platinum (Figure 4).

The Pt-to-C ratio for the reference catalysts 20% Pt XC-72R and HiSpec 3000 was 0.035 and 0.031, respectively, which means that the Pt content in both catalysts should be very similar due to the comparable area distribution of the PtNPs. According to the manufacturer's data, both materials contain 20 wt % of Pt. A similar Pt-to-C ratio of 0.033 was found for sample A coated with Pt using 50000 laser pulses, which suggests that this sample should be comparable to the reference materials in terms of Pt content. However, given the differences in PtNPs area distribution compared to the reference catalyst, an assumption based only on the XPS measurement may be incorrect. Using 60000 laser pulses in the case of sample B increased the Pt-to-C ratio to a value of 0.056. These results show that the PLD method enables the control of the amount of deposited Pt and suggests that the number of PtNPs should be higher compared to sample A. A surprisingly high Pt-to-C ratio of 0.087 was obtained for sample C, where Pt was deposited using only 10000 laser pulses on the synthesized C-11 material with a high degree of graphitization. This result can be explained by the fact that sample C has the most homogeneous layer of PtNPs with the smallest dimensions and dense distribution on the surface of carbon particles, which also correlates well with the TEM results (Figure 3). It also shows that the Pt-to-C ratio determined based on XPS measurements is inconsistent with the number of laser pulses used for Pt deposition on various carbon supports.

The high-resolution spectra of the Pt 4f band of material A and the reference catalyst 20% Pt XC-72R, along with the analysis of the oxidation state of platinum, are shown in Figure 4. The method of analysis used, its justification, and the Pt 4f band model have been described in detail in our earlier work [41]. The analysis showed that in both materials, Pt is in metallic Pt(0) form (Pt 4f_7/2_: peak position 71.29 ± 0.02 eV, FWHM 1.38 ± 0.03 eV) as well as in the oxidized forms PtO (Pt 4f_7/2_: peak position 72.17 ± 0.02 eV, FWHM 1.50 ± 0.01 eV), PtO_2_ (Pt 4f_7/2_: peak position 73.94 ± 0.02 eV, FWHM 2.40 ± 0.01 eV), and Pt(OH)_2_ (Pt 4f_7/2_: peak position 72.85 ± 0.02 eV, FWHM 1.58 ± 0.14 eV). In both cases, the metallic form of Pt dominates and constitutes 64.3% and 76.6% for material A and 20% Pt XC-72R, respectively (data on the graph). Among the oxides, the PtO form is dominant and constitutes 21.4% and 14.3% for material A and 20% Pt XC-72R, respectively. These result mean that the PLD-deposited Pt on the carbon support is oxidized to a higher extent than the reference Pt catalyst. Based on our experience with Pt deposited by PLD as thick continuous layers, slightly oxidized Pt layers can be deposited by the PLD method under vacuum conditions. Thus, we suspect that when Pt is deposited on a carbon support, it forms bonds with the oxygen atoms on the surface of the carbon particles. As a result, it can stabilize the position of the PtNPs on the surface of the carbon particles, which is essential for the stable long-time operation of the fuel cell.

Different values of the Pt-to-C ratio, which are proportional to the number of laser pulses used, were calculated based on the EDS measurement (Table 1). In the EDS technique, the signal is recorded from a larger volume of material (a pear-shaped area with a typical diameter of 2–5 µm). Due to the dimensions and structure of the material, it can be concluded that the EDS measurements should give a well-averaged quantitative result. Additionally, to determine the best estimate of the Pt-to-C ratio, EDS measurements were made on an area of 500 × 500 µm, which provided even better averaging. The Pt-to-C ratio was determined based on the atomic percentage of the elements.

The highest Pt-to-C ratios were obtained for the reference catalysts, 0.016 and 0.013 for 20% Pt XC-72R and HiSpec 3000, respectively. The samples fabricated using the PLD technique had a lower Pt-to-C ratio than the reference catalysts. The highest Pt-to-C ratio of 0.005 was obtained for sample B fabricated by deposition of Pt using the highest number of laser pulses. The value of the calculated ratio indicates that the amount of Pt by weight for sample B is approximately three times lower compared to the 20% Pt XC-72R. Taking into consideration that the reference catalyst contains 20 wt % Pt, the Pt mass fraction for sample B is estimated at approx. 6.3 wt %. For the remaining materials, the Pt-to-C ratio is lower and consistent with the number of laser pulses.

In conclusion, the XPS and EDS studies confirm that the developed method allows for the catalyst deposition on the surface of carbon particles. The results of XPS measurements show a clear correlation between the size of the PtNPs and the Pt-to-C ratio. Moreover, the deposition of Pt under vacuum conditions ensures that the oxidation of Pt is low as for the reference material (Figures 4b and 4c). This observation is confirmed by the almost identical shape of the Pt 4f band (the same position and width of the peaks) for sample A and the reference catalyst 20% Pt XC-72R (Table 1).

### RRDE measurements of ORR activity of Pt catalysts

**Figure 5:**
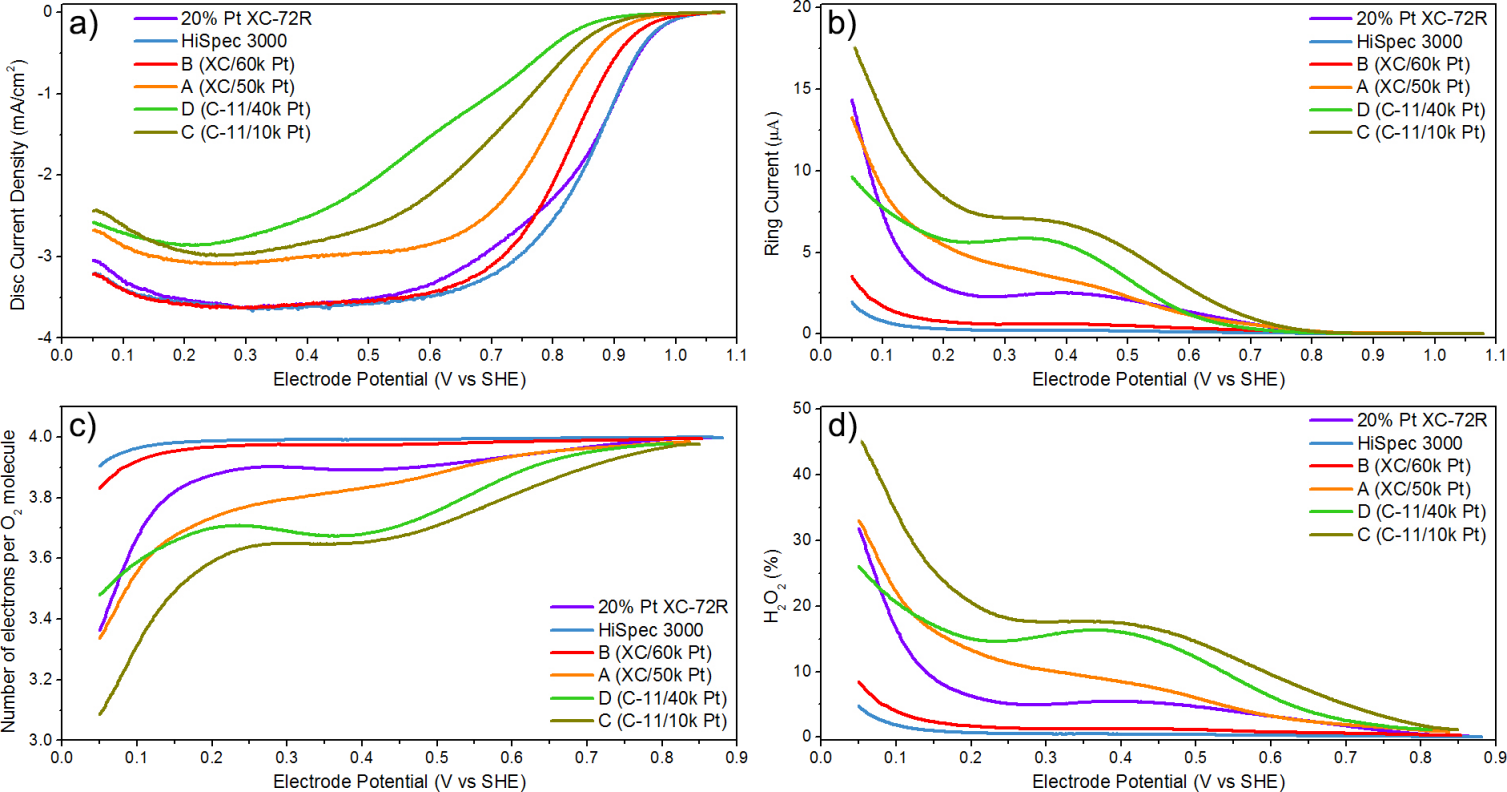
Curves recorded in the rotating disc electrode setup (at 900 rpm) for different Pt-based catalysts: (a) disc current density for the ORR, (b) corresponding limiting ring current at a fixed potential of 1.030 V, (c) number of electrons per O_2_ molecule formed during the ORR, (d) %H_2_O_2_ formed during the ORR. Curves (c) and (d) were calculated from the data in panels (a) and (b) using Equations 1 and 2, respectively, with η = 0.152 for 900 rpm. The thin film RRDE experiments were done in a four-electrode arrangement in 0.5 M H_2_SO_4_ solution oxygenated with pure O_2_ at 20 °C and 1 bar O_2_, with a potential scan rate of 5 mV/s.

The activity of the obtained Pt-based catalysts was initially assessed under well-defined rotating ring-disk electrode (RRDE) conditions. The ORR activity of Pt-based catalysts was evaluated in O_2_-saturated 0.5 M H_2_SO_4_ solution (Figure 5, Table 2, and Supporting Information File 1, Figure S3). Figure 5 allows for a quick comparison of the properties of the investigated catalysts. The number of electrons obtained in the oxygen reduction reaction (the number of electrons per O_2_ molecule) on electrodes made of the tested catalytic materials, and the resulting amount of hydrogen peroxide produced were determined based on polarization curves recorded using a rotating electrode with an RRDE ring [42]. The number of electron was calculated as:


[1] $$ne^{-}/{\text{O}}_{2}=\left(-4\cdot I_{\text{D}}\right)/\left(-I_{\text{D}}+I_{\text{R}}/\eta \right)$$

while the amount of hydrogen peroxide was calculated from the equation:


[2] $$\%{\text{H}}_{2}{\text{O}}_{2}=\left(2\cdot I_{\text{R}}/\eta \right)/\left(-I_{\text{D}}+I_{\text{R}}/\eta \right)\cdot 100\%$$

where *I*_D_ is the disc current, *I*_R_ is the ring current, and η is the electrode collection coefficient.

**Table 2:** Onset potentials (*E*_onset_), half-wave potentials (*E*_1/2_), and limiting current densities (LCD) at 0.2 V determined on the basis of RRDE electrochemical experiments performed for all Pt-based catalysts at 900 rpm, and maximum cell power density determined on the basis of Figure 7b below.

Catalyst	*E*_onset_ [V]	*E*_1/2_ [V]	LCD at 0.2 V [mA/cm^2^]	Maximum cell power density [W/cm^2^]

A (XC/50k Pt)	0.898	0.790	3.06	0.33
B (XC/60k Pt)	0.928	0.821	3.58	0.41
C (CMg/10k Pt)	0.880	0.720	2.93	0.06
D (CMg/40k Pt)	0.865	0.640	2.85	0.12
20% Pt XC-72R	0.968	0.860	3.52	0.57
HiSpec 3000	0.960	0.862	3.56	0.55

The results of RRDE measurements were compared for Pt catalysts prepared using the PLD method and commercial platinum catalysts containing 20 wt % platinum, 20% Pt Vulcan XC-72R and HiSPec 3000 carbon-supported materials. Both commercial catalysts were of high quality and yielded durable, homogeneous suspensions, which, after drying on the rotating electrode disk, formed tight and homogeneous layers over its entire surface. The layers of both catalysts adhered quite permanently to the carbon electrode. After the completion of the experiments, no significant loss of coverage occurred due to the centrifugal force of the rotating electrode. The oxygen reduction efficiency of both commercial catalysts is similar (Figure 5a, Table 2), but the catalyst HiSpec 3000 has a small advantage due to the lower percentage of hydrogen peroxide in the oxygen reduction products (Figure 5d). The fabricated catalysts of series A, B, C, and D also formed high-quality suspensions. However, the used C-11 carbon support affected the suspension quality of materials C and D as they tended to sediment after a longer period.

Based on the results of the disc current density measurement (Figure 5a), it can be concluded that materials C and D, made from the C-11 carbon support, show the weakest performance. This observation is also evidenced by the lowest onset (*E*_onset_ = 0.865 V) and half-wave (*E*_1/2_ = 0.640 V) potential values for material D (Table 2), limiting the current density and activities of the catalysts, assessed via RRDE experiments. Materials A, C, and D are characterized by a higher percentage of the two-electron reduction of oxygen in the mechanism than in the case of the commercial catalyst, which translates into a higher contribution of hydrogen peroxide in the reaction products (Figure 5d). Material C excels in this comparison, which is consistent with its composition, namely the lowest amount of platinum on the surface, which is responsible for the four-electron reduction of oxygen, and a significant fraction in the reaction of active carbon centers, which reduce oxygen in a two-electron process. The best performance was obtained using material B, prepared based on the XC-72R carbon support. It is evidenced by the highest onset (*E*_onset_ = 0.928 V) and half-wave (*E*_1/2_ = 0.821 V) potential values and good transportation properties represented by a limited current density (LCD) value on the same level as commercial catalysts (Table 2). The disc current density plot for material B coincides very well with the shape of the plot of the reference catalyst HiSpec 3000. It is also worth noting the very low concentration of hydrogen peroxide in ORR products of material B (Figure 5d). The concentration of hydrogen peroxide is only slightly higher than for the best catalyst in this respect, HiSpec 3000, and several times lower than for the second reference catalyst, 20% Pt XC-72R. This result is very interesting because both catalysts (material B and 20% Pt XC-72R) are based on the same XC-72R carbon support. A smaller fraction of H_2_O_2_ in ORR products means a greater energy benefit of the reaction in fuel cells, because material B gains more electrons from the reaction (a value close to 4). Material B, therefore, clearly outperforms the reference 20% Pt XC-72R catalyst. Additional investigations are needed to determine the cause of the apparent differences.

Before performing ORR experiments in the RRDE system, cyclic voltammetry curves in N_2_-saturated 0.5 M H_2_SO_4_ were recorded. In cycling voltammetry experiments, materials A and B show signals characteristic of the platinum nanoparticle surface (Supporting Information File 1, Figure S4). With the increase in the number of laser pulses, the signal for platinum on the voltammograms increases, which confirms the increase in the amount of deposited Pt. In the case of material B, along with an increase in the number of electrode polarization change cycles, a process of active platinum surface unfolding is observed. This observation is evidenced by the increase in current signals registered in the electrode potential areas characteristic of platinum surface processes, the processes of oxidation and reduction of hydrogen at low potentials, and the processes of oxidation of the platinum surface and reduction of surface oxide occurring at potentials higher than 0.6 V. Materials C and D have a residual platinum signal indicating the presence of platinum in the low-potential region where the adsorbed hydrogen oxidation and reduction reactions take place. These results indicate that a small amount of platinum was deposited on the carbon support. Considering the amount of deposited platinum, material C is the least active, and the shift of the reduction half-wave potential reaches 100 mV. In the case of material A, the shift in the reduction half-wave potential is about 50 mV. The durability of fabricated catalysts in RRDE tests is, after all, proportional to the amount of deposited platinum.

### Efficiency of manufactured catalysts in a fuel cell system

**Figure 6:**
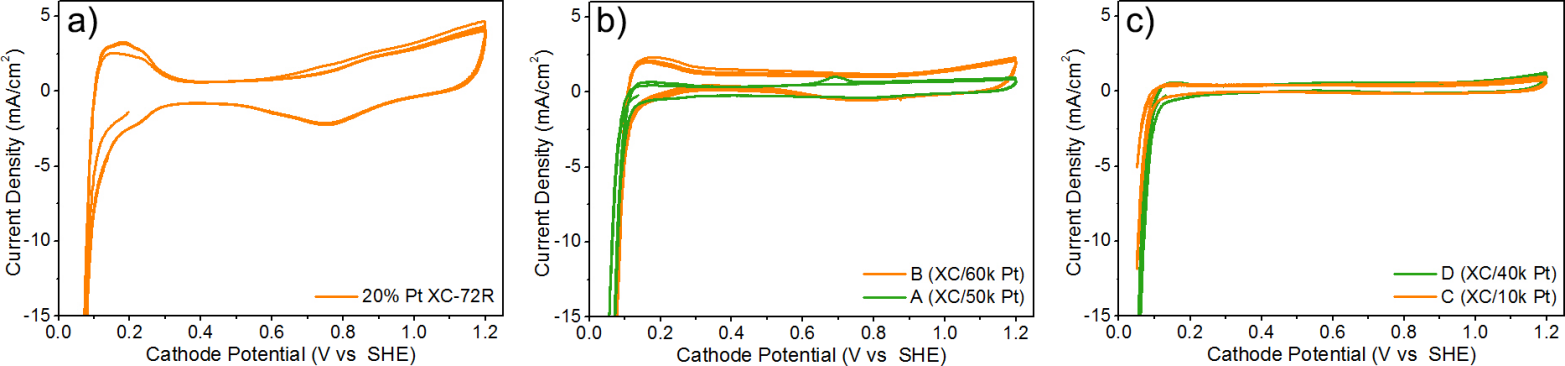
Cyclic voltammetry curves for Pt-based catalysts: (a) reference catalyst 20% Pt XC-72R, (b) materials A and B with Pt deposited on Vulcan XC-72R, and (c) materials C and D with Pt deposited on synthesized carbon material C-11. Curves were recorded at a scan rate of 0.01 V/s in a fuel cell with humidified N_2_ (cathode) and H_2_ (anode) gas feeds at ambient temperature and pressure. Each curve consist of four consecutive voltage cycles.

Fabricated materials A, B, C, and D were used to make electrodes to investigate the efficiency of the fabricated Pt-based catalysts in a membrane electrode assembly (MEA) of PEMFCs fed with H_2_/air. The materials with Pt deposited on Vulcan XC-72R (samples A and B), like commercial catalysts, formed a good quality catalyst layer on the Nafion membranes. However, we encountered some issues during electrode preparation from the materials based on a synthesized carbon support C-11 (samples C and D) that were related to their wettability. After transferring approximately half of the material to the electrode, the catalyst layer obtained was not evenly wetted with the fresh ink. Adding a few drops of isopropanol did not improve the painting conditions. However, as a result, a mechanically robust layer was obtained, which did not fall off the electrode spontaneously. Therefore, the more graphitized C-11 carbon material used in Pt-based catalysts is more difficult to apply on Nafion membranes.The cyclic voltammetry curves measured in the MEA setup for all prepared samples are shown in Figure 6. The CV curves of the reference material used to prepare the anodes of all cells are almost identical, which proves the high repeatability of the cell preparation method. The CV curves of one of the cell anodes are shown in Figure 6a. They have well-developed characteristics of a platinum–carbon material. This confirms their quality, which does not differ from high-quality catalysts of this type (such as from Johnson Matthey, ETEK). The CV curves of the catalysts fabricated by PLD indicate a worse development of the platinum surface on the carbon (Figure 6b,c). This observation is proven by less visible signals in the range of low potentials at which the processes of hydrogen reduction and oxidation take place and the lack of an oxygen reduction signal occurring on platinum at a potential of about 750 mV. The presence of Pt on the electrode surface is proven by the hydrogen evolution current registered at a potential of about 50 mV. Based on the comparison of the CV curves, the best catalyst from the point of view of Pt surface development is sample B, while the worst is sample C. These results are as expected and correlate well with the amount of Pt deposited by PLD.

**Figure 7:**
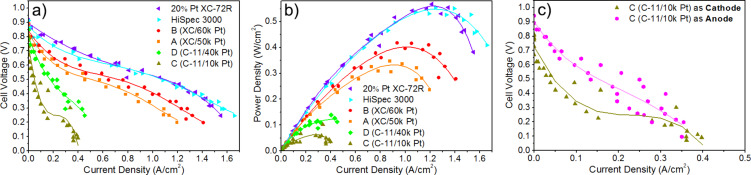
The results of the tests performed in PEMFCs supplied with H_2_/air: (a) steady-state polarization curves for the materials being tested as a cell cathode, (b) steady-state power density curves for the materials being tested as a cell cathode, and (c) steady-state polarization curves for material C being tested as a cell cathode and anode. The temperature of cell and humidifier was 80 °C, the backpressure at cathode and anode was 2.0 bar. The cathode was fed with air at 500 std cm^3^/min, and the anode was fed with H_2_ at 100 std cm^3^/min.

The results of performance tests of Pt-based catalysts conducted in a membrane electrode assembly are shown in Figure 7. Polarization curves shown in Figure 7a are used to assess the influence of cell construction on the value of activation, resistance, and transport losses [33]. These losses result accordingly from the necessity to overcome the activation barriers of electrode reactions, the non-zero internal resistance of the cell, and resistance of diffusion and convection of the substrates and products of the electrode reactions. All losses coincide at each point of the polarization curve. However, their contribution differs depending on cell voltages. At high cell voltages, a contribution is dominated by activation losses; at intermediate voltages, it is dominated by resistance losses; and at low voltages by transport losses.

The polarization curves of the cells obtained when fed with pure hydrogen and air provide information about the operation of the catalysts produced (Figure 7a). Each polarization curve point corresponds to the stationary state of the cell reached after 60 s from the stabilization of the voltage at the cell terminals [33]. The curves were determined based on two cycles in which the current consumption from the cell was increased in the first cycle, while in the second cycle, it was reduced, returning to the open-circuit voltage. The marked continuous line corresponds to the approximation of the measurement points by third- or fourth-degree polynomials. The dispersion of the measurement points visible for the individual curves results mainly from the nonuniform water transport in the cell (water management). Water enters the cell as water vapor with together the humidified gases and forms during the reactions. It condenses in the catalytic and diffusion layers. The microscopic water droplets must leave the MEA and the cell, but in some cases, they are temporarily trapped in the diffusion layer or the flow fields. The presence of water results in a temporary decrease of the current due to the transport resistance increase, and the current increase when the water leaves the cell.

Considering the polarization curves and the maximum power density of the cells (Table 2, Figure 7), material B shows the highest efficiency in the cell, while material A is slightly behind it. The open-circuit voltage of the cell, corresponding to the cathode potential under these conditions, is at the level of 880 mV for both materials. It is slightly lower than the voltage of the reference cell, which was approx. 915 mV. The best polarization curve recorded for cell A is about 50 mV lower than that for cell B. The polarization curve recorded under similar conditions on the reference cell (20% Pt XC-72R) is better in the entire potential range and raised by about 100 mV compared to the best curve of cell B. Comparing the curves for materials A and B with the curve for the reference catalyst shows that higher activation losses characterize materials A and B. However, resistance and transport losses observed for materials A and B are comparable to losses for the reference catalyst. Moreover, activation losses are higher for material A with less deposited Pt.

Materials C and D stand out significantly from the other samples. The cathode potential in the cell's open circuit (approx. 800 mV) is too low, considering that the catalyst contains platinum. With such a low open-cell voltage, it is impossible to obtain high power. The cell must be highly biased to a voltage of 200 mV to start producing electricity and, consequently, enough water to moisten the membrane. A self-propelling mechanism is created, but there are a low current, insufficient water in the diaphragm, a high internal diaphragm resistance, and a power drop. The attempt to support the cell by increasing the cathode hydration (to about 125% RH) did not improve the cell performance at low load and did not translate into higher cell power. These cells are characterized by very high activation losses and higher resistance and transport losses than the reference cells.

Material C, characterized by the lowest efficiency, was also tested as an anode for the cell. An efficiency improvement could be expected due to the ease with which hydrogen reaches the active centers of platinum. As a result, the open-circuit voltage should increase and allow for a higher current density in the initial range of the polarization curve, translating into a higher course of the curve throughout the polarization range. The results obtained (Figure 7c) confirmed the assumption about the increase in performance of this material as an anode catalyst for hydrogen fuel cells. The improvement in operation is visible in the catalytic range of the polarization curve, as evidenced by the appearance of measurable current values with low cell polarization. An increase in the open-circuit voltage of the cell was also observed. The activation, resistance, and transport losses also determine the maximum power densities obtained from the cells (Table 2, Figure 7b). The highest power density values of 0.57 W/cm^2^ and 0.55 W/cm^2^ were obtained for cells with a cathode made of 20% Pt XC-72R and HiSpec 3000 materials, respectively. Among the materials produced by the PLD method, the highest power density of 0.41 W/cm^2^ was obtained for material B containing the largest amount of deposited Pt. In addition, as proved by the EDS measurements, such a result was obtained for a material containing three times less platinum than the reference 20% Pt XC-72R. Based on the above results, the utilization of Pt deposited by the PLD method is estimated to be approximately twice as high compared to the reference material. The lowest values of the maximum power density were obtained for samples C and D. Apart from the fact that these materials were produced using the smallest number of laser pulses during PLD coating, the influence of the carbon support itself is also evident. The more graphitized carbon material is a higher barrier to be overcome for the gases and the water generated in reactions occurring in the cell.

## Conclusion

The fabrication of an efficient catalyst based on carbon particles with low Pt loading was successfully achieved by using the PLD method. The structural characterization of fabricated catalysts by HAADF, EDX, and TEM showed a greater uniformity and a higher dispersion of the PtNPs on the carbon supports compared to the reference catalysts. However, only in the case of catalysts based on the commercial carbon support Vulcan XC-72R maximum cell power densities comparable to reference catalysts were obtained. The best performing material fabricated in our studies, Pt deposited on Vulcan XC-72R with 60000 laser pulses (material B), had a power density value reaching over 70% of the power density values of the reference catalysts. However, it had a three times smaller amount of Pt than the reference catalyst, 20% Pt Vulcan XC-72R.

The results prove that it is possible to replace the chemical methods of PtNP deposition on carbon supports, which can be used as a catalyst in PEMFCs, with the PLD method. In addition, our studies show that with a developed particle mixer, PLD is no longer limited to the deposition of thin metal layers on flat solid substrates but can be applied to substrates in the form of particles. The particle mixing system developed in combination with PLD can also be used to deposit different materials on various types of particles for other applications. The method developed still requires additional investigations on production scalability, repeatability, and automation of the Pt deposition process. It is also crucial to test the chemical stability of the material in the working polymer fuel cell system over a long period.

## Experimental

### Materials

Two carbon materials with similar specific surface area, namely Vulcan XC-72R carbon black powder (purchased from Fuel Cell Store, United States) and a synthesized carbon material (C-11) with a high graphitization degree, were used as carbon support. The carbon material C-11 was synthesized in a strong exothermic redox reaction (self-propagating high-temperature synthesis, SHS) between pulverized anhydrous calcium formate and magnesium powder (all reagents purchased from Sigma-Aldrich, United States).

### Pulsed laser deposition of platinum on the carbon supports

The platinum catalyst was deposited on the carbon support using a pulsed laser deposition (PLD) system equipped with an ArF excimer laser (LPX Pro 305, Lambda Physik). The following parameters characterize the laser used in the experiment: λ = 193 nm, *E* = 600 mJ, τ = 15–25 ns. The two-stage pumping system used allows for an oil-free vacuum to be achieved. The catalyst was deposited from a 20 × 20 × 2 mm rotating platinum target with a purity of 99.95% (Kurt J. Lesker, United Kingdom).

The laser beam was focused on the target at an incident angle of 45°. The distance between the target and the bottom of the carbon support container was constant and equal to 65.0 ± 0.5 mm. The laser focus measured area on the target surface was 1.28 ± 0.14 mm^2^, and the estimated energy of the laser pulse on the target surface for all experiments was 302 ± 3 mJ. The laser fluence on the target surface, calculated on this basis, was 23.6 ± 2.4 J/cm^2^. All depositions were performed with the exact laser repetition rate of 5 Hz and at room temperature. Other parameters of the deposition process, different for consecutive samples, are presented in Table 1.

**Figure 8:**
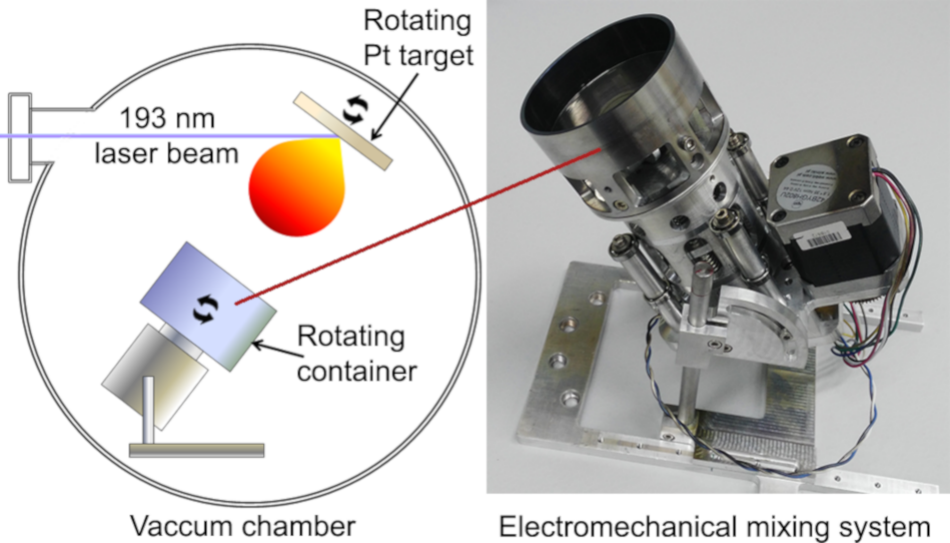
A schematic diagram of the PLD experimental setup (on the left) and a photo of the developed carbon support mixing system (on the right).

An electromechanical system for continuous carbon support mixing during deposition was developed to obtain a uniform deposition of platinum on the carbon support (Figure 8). A constant, experimentally selected mass of carbon material was placed in a cup oriented at an appropriate fixed angle to the plasma stream. During platinum deposition, the cup was rotated at a constant speed and put into vibration with an experimentally selected frequency and amplitude.

### Structure, morphology, and chemical composition

The carbon material quality was evaluated based on Raman spectra. Raman measurements were performed using a commercial Renishaw InVia Reflex Raman microscope equipped with an EMCCD (1600 × 200 pixels) detector (Andor Technology Ltd, Oxford Instruments, United Kingdom). The Raman signal was acquired using laser radiation at 633 nm. The laser beam was directed to the sample through a 50× (N.A. = 0.75) objective lens. For the lens used, the diameter of the measuring point from which the Raman signals are recorded is approximately 2 μm. The instrument's wavelength was calibrated using an internal silicon wafer, and the spectrum was centered at 520.5 cm^−1^. The measurement parameters were an acquisition time of 10 s and ten accumulations at each point. The excitation laser power on the sample was constant for all samples and was approximately 0.68 mW. The Raman spectra presented are averaged spectra calculated from ten spectra registered at random points on the samples. Data processing was performed using WiRE 5.4 software.

The specific surface area (*S*_BET_) of the carbon materials was determined based on N_2_ adsorption–desorption measurements performed at liquid nitrogen temperature using an Autosorb IQ analyzer (Quantachrome Instruments – Anton Paar, United States). The samples were outgassed at 250 °C for 10 h prior to analysis. The Brunauer–Emmett–Teller specific surface area (*S*_BET_) was calculated from the N_2_ adsorption isotherms, considering the IUPAC recommendations [39]. The total pore volume (*V*_0.99_) was estimated from the N_2_ volume adsorbed at a relative pressure (*p*/*p*_0_) of approx. 0.99. The volume of micropores (*V*_mi_) was estimated using the Dubinin–Radushkevich (DR) model.

The morphology of the carbon material was examined using a scanning electron microscope (SEM) equipped with energy-dispersive spectroscopy (EDS). SEM measurements of the samples were carried out using a Quanta 3D FEG microscope (FEI, United States). The surface imaging and EDS measurements were done at an accelerating voltage of 20 kV and identical work parameters of the EDS detector.

The quality of platinum coating on the carbon material was assessed based on images made using a transmission electron microscope (TEM) equipped with an energy-dispersive X-ray spectrometer (EDX). TEM measurements were carried out at the Biological and Chemical Research Centre, University of Warsaw, Poland, on the TALOS F200X (Thermo Fisher Scientific, United States) equipped with a four-detector windowless Super X-EDS system. The EDX measurements were performed in STEM (scanning transmission electron microscopy) mode with a spatial resolution of 160 pm using the HAADF (high-angle annular dark-field) detector. Statistical analysis was performed on the HRTEM images using the Digimizer software. About 200 particles were measured to assess their mean area and area distribution.

For the measurement of the chemical composition of the prepared platinum-coated carbon support, X-ray photoelectron spectroscopy (XPS) was used (Prevac, Poland) with an R3000 VG analyzer (Scienta, Sweden) and an X-ray source with an Al Kα anode (Prevac, Poland) emitting X-rays with a photon energy of 1486.6 eV. The analysis of the XPS spectra registered was performed using CasaXPS software.

### Electrochemical measurements (RRDE and single fuel cell testing)

Measurements of the electrochemical properties of the samples of catalytic materials prepared were performed on a fuel cell test stand at the Łukasiewicz Research Network - Mościcki Industrial Chemistry Research Institute (ICRI) in Warsaw. In the first stage, the efficiency of electrochemical oxygen reduction was measured using the rotating disc-ring electrode (RRDE) technique. The measurements were conducted in a four-electrode electrochemical cell using a RRDE (Pine Instruments, United States) with a glassy carbon (GC) disc electrode of 0.1642 cm^2^ surface, driven by a CHI 700C bipotentiostat (CH Instruments, United States). A solution of 0.5 M H_2_SO_4_ was used as the base electrolyte. A mercury sulfate electrode Hg/HgSO_4_/1 M H_2_SO_4_ (potential 600 mV), and a gold metal sheet were used as reference and counter electrodes, respectively. All potentials were converted to the standard hydrogen electrode (SHE) scale. The catalyst materials studied were suspended as ink in deionized water (double distilled water + purified on Millipore filters), isopropanol (IPA), and a 5 wt % suspension of Nafion™ (1100 e.w., Ion Power Inc., United States) by ultrasonic mixing. The ratio of the ionomer to the studied Pt-based catalyst mass in a dry electrode was 0.1. The disc-ring electrode surfaces were polished and checked for purity before applying the catalyst layer by cyclic voltammetry in a 0.5 M H_2_SO_4_ solution deoxygenated with N_2_ (purity 5.0). The catalyst ink was mixed and suspended using an ultrasonic cannon (750 W, Cole-Parmer, United States), impulse sonication with 150 W energy for approximately 1 min. A 20 µL drop of the suspension was placed with a micropipette on the disc of the spinning electrode. The electrochemical reduction of oxygen was studied in a sealed vessel using a four-electrode system in a solution of 0.5 M H_2_SO_4_ continuously oxygenated with oxygen 5.0 from the cylinder while maintaining an oxygen cushion over the base electrolyte solution.

In the next stage, the efficiency of the carbon supports coated with Pt as a catalyst in the fuel cell was investigated in a membrane electrode assembly (MEA) of PEMFCs fed with H_2_/air. The test samples coated with Pt by the PLD method were used as the cathode catalyst of the cell. The anode used was a layer of commercial Pt-based catalyst containing 20 wt % Pt deposited on Vulcan XC-72R (Fuel Cell Store, United States) subjected to the same method as the cathode layer by painting to the opposite side of the Nafion membrane (Nafion™ 112, DuPont, United States). The anode and cathode ink were prepared using an ultrasonic cannon (750 W, Cole-Parmer, United States) impulse sonication with 150 W energy for approximately 1 min by mixing Pt-coated carbon material, 5% alcohol suspension of Nafion (1100 e.w., Ion Power, United States) and double distilled water. The suspension was prepared in an airtight tube cooled with ice. The Nafion solution was applied in an amount needed to maintain approximately 30 vol % of dry Nafion in the catalytic layer. As a result, the catalyst was deposited on the cathode and anode surfaces in an amount of 0.2 mg Pt per cm^2^. The ink on the surface of the membranes was applied by brush painting at a temperature of approx. 65 °C. The active geometric surface area of the cathode and anode was 5 cm^2^. As a gas diffusion layer (GDL) on both sides of the MEA, the ELAT LT2500W carbon fabric (E-Tek) was used. The reference fuel cell was prepared the same way as the tested cells, except that the cathode was made of 20% Pt Vulcan XC-72R catalyst, while the anode was made of 20% Pt HiSpec 3000 catalyst.

MEA tests were carried out on a single-cell fuel cell assembly with a single serpentine flow field on both electrodes. The assembled cells were fed with ambient air supplied by an oil-free compressor and H_2_ 5.0 (and/or N_2_). All the fuel cell tests were recorded using a fuel cell station (150 W, Fuel Cell Technologies Inc., United States). This system controls the operating conditions of the fuel cell (cell temperature, gas flows, humidity, and pressure of anode and cathode gases). Cyclic voltammetry curves (CVs) were recorded with a SI1287 potentiostat–galvanostat (Solartron Instruments, United Kingdom). All electrochemical experiments were conducted at room temperature (ca. 25 °C).

## Supporting Information

File 1 Additional figures.
