# Supplementary material for: A novel approach to pulsed laser deposition of platinum catalyst on carbon particles for use in polymer electrolyte membrane fuel cells
Source: Beilstein J Nanotechnol. 2023 Feb 2;14:190–204. doi: 10.3762/bjnano.14.19 (PMC9907015; doi:10.3762/bjnano.14.19)
Supplement: File 1 — Additional figures. [file Beilstein_J_Nanotechnol-14-190-s001.pdf]

BEILSTEIN JOURNAL OF NANOTECHNOLOGY

Supporting Information

for

A novel approach to pulsed laser deposition of platinum catalyst on carbon particles for use in polymer electrolyte membrane fuel cells

Bogusław Budner, Wojciech Tokarz, Sławomir Dyjak, Andrzej Czerwiński, Bartosz Bartosewicz and Bartłomiej Jankiewicz

Beilstein J. Nanotechnol. **2023,** *14,* 190–204. doi:10.3762/bjnano.14.19

Additional figures

License and Terms: This is a supporting information file under the terms of the Creative Commons Attribution License (https://creativecommons.org/licenses/by/4.0). Please note that the reuse, redistribution and reproduction in particular requires that the author(s) and source are credited and that individual graphics may be subject to special legal provisions.
The license is subject to the *Beilstein Journal of Nanotechnology* terms and conditions: (https://www.beilstein-journals.org/bjnano/terms)

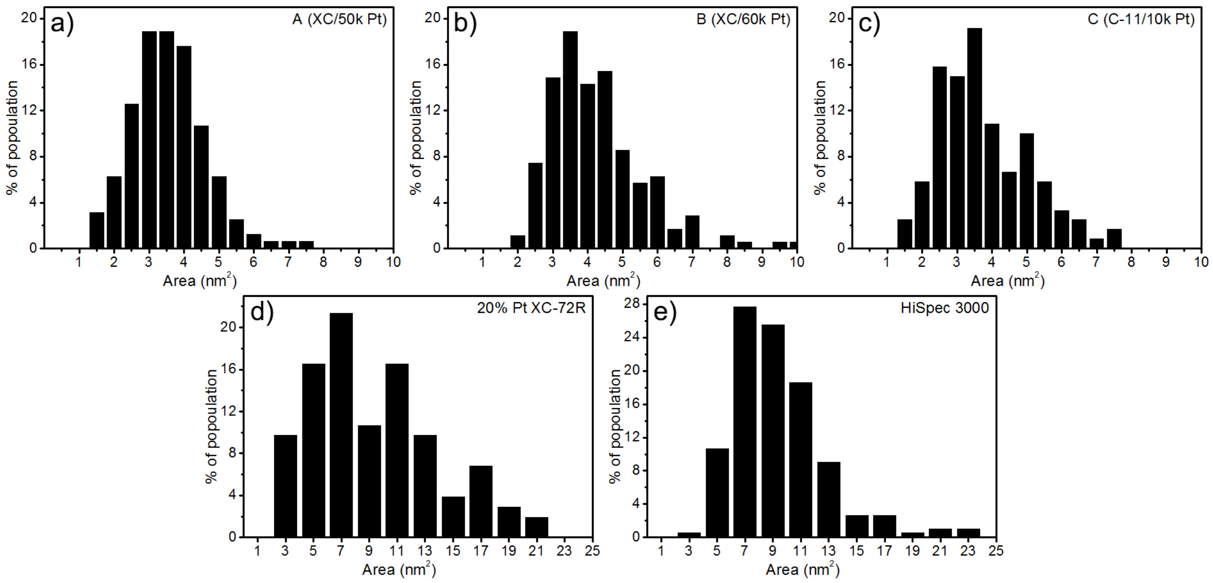

Figure S1: Statistical analysis of Pt nanoparticles area distribution based on the HRTEM images.

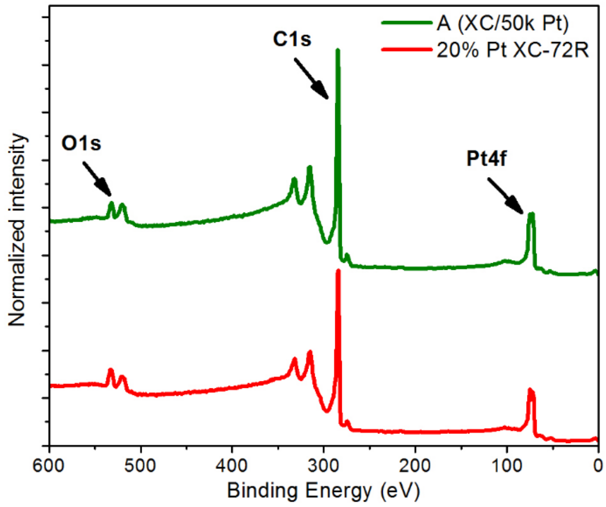

Figure S2: Low-resolution XPS spectra of the reference catalyst 20% Pt XC-72R (red line) and sample A (green line) prepared using the PLD method characterized by a similar Pt-to-C ratio.

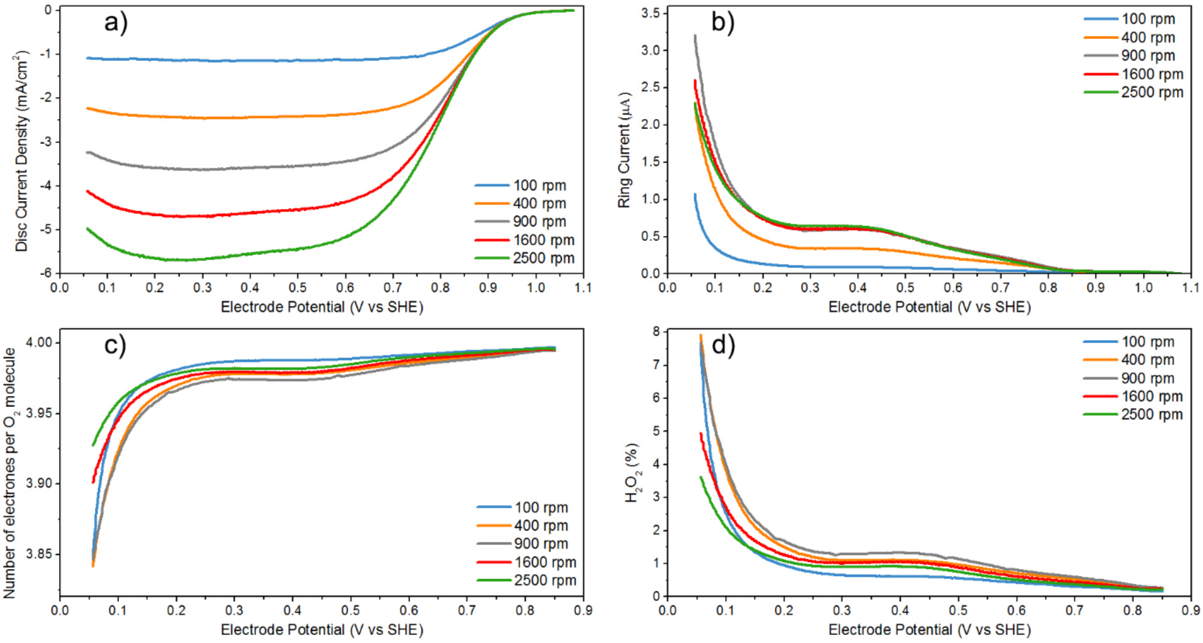

Figure S3: ORR curves recorded for sample B (XC/60k Pt) in the rotating disc electrode setup (at 100, 400, 900, 1600, and 2500 rpm), deposited on the GC disc electrode: (a) disc current density for the ORR, (b) corresponding limiting ring current at a fixed potential of 1.030 V, (c) a number of electrons per O_2 molecule formed during the ORR, (d) % H_2O_2 formed during the ORR. Curves (c) and (d) are calculated from the data in panels (a) and (b) using Equations 1 and 2, respectively, with η = 0.152 for 900 rpm. The thin film RRDE experiments were carried out in a four-electrode arrangement in 0.5 M H_2SO_4 solution oxygenated with pure O_2 at 20 °C and 1 bar O_2, with a potential scan rate of 5 mV/s.

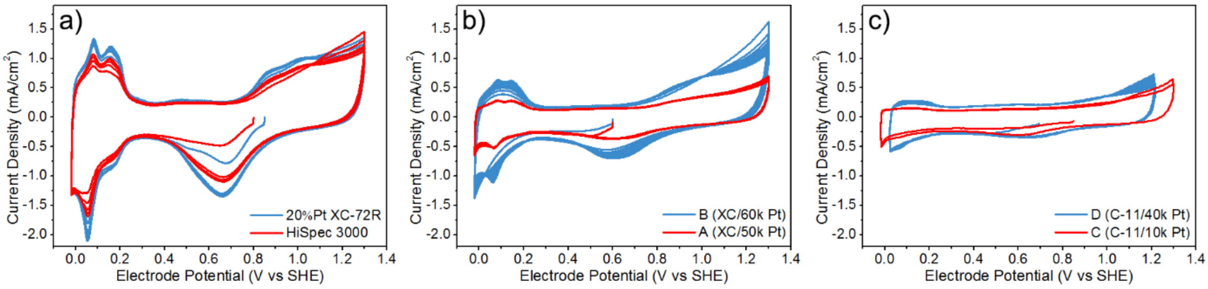

Figure S4: Cyclic voltammetry curves recorded in the rotating disc electrode setup for Pt-based catalyst deposited on the GC disc electrode: (a) reference catalyst 20% Pt XC-72R and HiSpec 3000, (b) materials A and B with Pt deposited on Vulcan XC-72R, (c) materials C and D with Pt deposited on synthesized carbon material C-11. Curves were recorded in N_2-saturated 0.5 M H_2SO_4 solution (1 bar N_2 pressure, 20 °C, potential scan rate 50 mV/s).
